# Increasing the capacity of community managed organisations to provide chronic disease preventive care to people with a mental health condition: protocol for a cluster-randomised controlled trial

**DOI:** 10.1186/s12889-025-25089-7

**Published:** 2025-12-17

**Authors:** Sophie Love, Caitlin Fehily, Ama Ampofo, Jade Ryall, Fadzi Marasha, Joanna Latter, Simone Lodge, Belinda Jackson, Elizabeth Campbell, Christopher Oldmeadow, Olivia Wynne, Mark Orr, Sumathi Govindasamy, Jenny Bowman

**Affiliations:** 1 School of Psychological Sciences, College of Engineering, Science and Environment, The University of Newcastle, Callaghan, NSW Australia; 2 Population Health, Hunter New England Local Health District, Wallsend, NSW Australia; 3 Population Health Research Program, Hunter Medical Research Institute, New Lambton Heights, NSW Australia; 4 Flourish Australia, Sydney, NSW Australia; 5 International Centre for Future Health Systems, UNSW Medicine & Health, University of New South Wales, Sydney, NSW 2052 Australia; 6 School of Medicine and Public Health, College of Health Medicine and Wellbeing, The University of Newcastle, Callaghan, NSW 2308 Australia; 7 Monash University Malaysia, Subang Jaya, Malaysia

**Keywords:** Mental health services, Community managed organisation, Preventive care, Smoking, Nutrition, Alcohol, Physical activity

## Abstract

**Background:**

Comprehensive and routine provision of preventive care for modifiable risk factors within mental health community managed organisations (CMOs) is variable.

**Methods:**

This protocol describes a planned cluster-randomised controlled trial to support the delivery of such care within a large Australian CMO. A model of care and implementation support strategies will be co-designed and delivered across six months, with 12 CMO sites randomised to intervention or control groups. The model of care referred to as ‘The Live Well and Flourish Framework’ will guide staff in intervention sites through support conversations about modifiable risk factors (stopping or reducing smoking/vaping, healthy eating, limiting alcohol, and being physically active), supported through implementation strategies delivered throughout the intervention. The primary outcome is the proportion of people accessing CMO services who report being connected to health behaviour change support and resources for ‘Living Well Activities’. The resources required to develop and implement the model of care and implementation strategies will inform a cost-effectiveness evaluation of the intervention.

**Discussion:**

Findings from this trial will inform future care delivery and initiatives within CMOs to provide support for modifiable chronic disease risk factors.

**Trial registration:**

Prospectively registered with the Australian and New Zealand Clinical Trials Registry (ACTRN12625000322437) on 17/04/2025.

**Supplementary Information:**

The online version contains supplementary material available at 10.1186/s12889-025-25089-7.

## Background

Both internationally and in Australia, having a mental health condition is associated with greater chronic disease morbidity and mortality; contributing to a global median gap in life expectancy of 10 years between those with a mental health condition and the general population [1, 2]. International research consistently reports that people with a mental health condition are 1.4 to 2 times more likely to experience chronic conditions such as obesity, diabetes, and cardiovascular disease than the general population [3]. These higher rates of chronic disease are contributed to by high prevalence of modifiable risk factors, including four key factors: smoking, poor nutrition, excess alcohol consumption, and physical inactivity [3].

In Australia, the role of mental health services in providing preventive care to support physical health, including support for changing risk factors, is increasingly acknowledged [4, 5]. One such setting is in community managed organisations (CMOs): non-government and non-profit organisations. These organisations are a key part of the mental health sector, with the CMO workforce representing 25% of the total mental health workforce [6]. They offer a range of services, including assistance with accommodation, employment, education, leisure, and counselling services [7, 8]. These organisations often employ recovery-oriented, person-led, trauma informed practices and have a focus on the overall wellbeing of the people they support [7–9]. As such, CMOs have been identified as an opportune setting to deliver preventive care and lifestyle interventions [10, 11].

Research indicates that although CMOs are typically providing preventive care as part of current practice, it may not always be provided in a routine or comprehensive manner. A 2018–2019 survey of NSW CMO leaders (*n* = 76), found that over 80% provided preventive care (defined as brief interventions to identify and address health risks) for at least one risk factor, (e.g., smoking, nutrition, alcohol, physical activity, or sleep), however, offers of preventive care for all risk factors was much lower (16–57%) [12]. Additionally, the type and quality of preventive care offered has been reported to vary [13]. A survey of staff from one of the largest CMOs in the states of NSW and Queensland (*n* = 174) measured staff-reported provision of preventive care, which included asking about health risk factors, offering advice and referring to another service (AAR Framework) [14] as well as assistance to support changes (from the 5As Framework) [15]. This study assessed what proportion of staff were providing ‘optimal’ support, defined as providing preventive care to at least 80% of people they support for four risk factors: tobacco smoking, inadequate fruit and vegetable consumption, harmful alcohol consumption and physical inactivity. Optimal support was reported by staff most commonly for ask (26%) and advice (28%), with less providing assistance (13%) or referrals to follow-up services or health professionals (12%). Overall, across the care elements, support was typically more frequently provided for physical activity and least for alcohol [13].

Several barriers to providing preventive care have been identified in this setting, including a lack of staff training and guidelines [12, 16]. In the aforementioned survey of NSW CMO leaders, 70% of CMOs provided staff training for at least one risk factor, with about half offering training for each of the four factors (52–55%). Only 45% provided staff with guidelines about the delivery of preventive care for at least one risk factor. When training and guidelines were offered, staff were more likely to ask and assist the people they support with making changes [12]. In the survey of CMO staff, it was found that more than 80% of staff agreed that provision of preventive care would be beneficial for mental and physical health. However, around a quarter of staff stated it was challenging to provide care for fruit and vegetable consumption and smoking [16].

Research to increase the delivery of preventive care specifically for CMO settings is limited, with most research of a lower evidence level (e.g. pre-post evaluations) [17]. One rapid review evaluated the current evidence for models of delivering preventive and physical health interventions in CMOs [17]. Eight studies were identified across multiple countries, including Australia and the United States, addressing chronic disease management (*n* = 2), multiple risk factors (*n* = 5), and oral health (*n* = 1) [18–26]. The evidence base comprised three randomised controlled trials and five pre–post evaluations, with follow-up ranging from six months to two years. Five studies demonstrated significant improvements in care provision or consumer physical health outcomes [18–20, 23, 26]. Interventions varied widely with studies using: (i) peer-led self-management programmes [18, 19], (ii) enhanced care providers’ capacity [23], (iii) embedded nursing support for preventive screening [20] (iv) combined nurse-led staff training with wellness coaching [26]. All studies utilised multiple implementation strategies such as staff training, tailored resources, and site champions. The review highlighted a need for continued research to co-develop appropriate and effective interventions to support preventive care delivery in CMOs.

To the authors knowledge, since this review there has been one study conducted in a CMO setting to evaluate the effectiveness of a practice change intervention to implement a model of care for the four key risk factors; smoking, nutrition, alcohol and physical activity [27]. The study was an Australian non-randomised controlled pilot trial. The practice change intervention was co-developed with CMO staff and used implementation strategies to support delivery of a model of care (informed by AAR): conversations, assistance and connections (to health behaviour change services or resources) (CAC). The implementation strategies included ‘healthy conversation skills’ (HCS) training [28] and educational materials. These strategies were delivered as a three-month support package for staff. Compared to a usual care control group, findings indicated improvements in CMO staff perceived barriers and facilitators to care, such as confidence, as well as to the perceived individual and organisational abilities to provide support across all four risk factors (except for organisational ability to provide support for physical activity behaviour). While this pilot study provides preliminary evidence of the potential effectiveness of a CMO practice change intervention to increase delivery of preventive care for multiple risk factors, this trial did not measure provision of care as an outcome. The study did not consider consumer perspectives in the co-development and outcomes measured [29, 30]. Ongoing investigation is needed to establish co-developed models of care and implementation strategies with both staff and consumers, to ensure they are acceptable and appropriate for the CMO setting. This includes ensuring alignment of such interventions with the non-clinical nature of support provided by CMOs, including adopting person-directed and strength-based approaches within a recovery framework [8, 17]. Economic evaluation is recommended as a method to assess the efficiency and value of health care, such as preventive care interventions [31, 32]. However, there are few economic evaluations of implementation interventions in mental health settings [33, 34] and to the authors knowledge there have been no economic evaluations of preventive care interventions in community managed organisations specifically.

While mental health CMOs are incorporating preventive care as part of their current practice, there is a need for consistent and comprehensive approaches. Further research determining appropriate and effective approaches to implementing routine preventive care into the CMO setting, as well as economic evaluation of the value of these initiatives are needed. This paper describes the protocol for a trial which will be undertaken in collaboration with a CMO to assess the effectiveness of a co-developed practice change intervention (inclusive of a model of care for preventive care delivery and implementation strategies) on the provision of support for modifiable risk factors (stopping or reducing smoking/vaping, healthy eating, limiting alcohol, and being physically active), referred to hereafter as ‘Living Well Activities’.

## Methods

### Study design and setting

A cluster randomised controlled trial with two parallel groups will be conducted within one large mental health CMO (Flourish Australia). The unit of randomisation will be sites, with 12 sites participating across three regions (Hunter and New England, Queensland, South Australia; as defined by Flourish Australia). In the 2023/2024 financial year, Flourish Australia supported 8,975 people [35]. The type of services provided included support for housing, employment, social opportunities, as well as access to services such as The National Disability Insurance Scheme (NDIS), Headspace and other clinical services. Approximately 36% of the service delivery team are peer workers, using their own mental health lived experience to inform their practice [35].

**Fig. 1:**
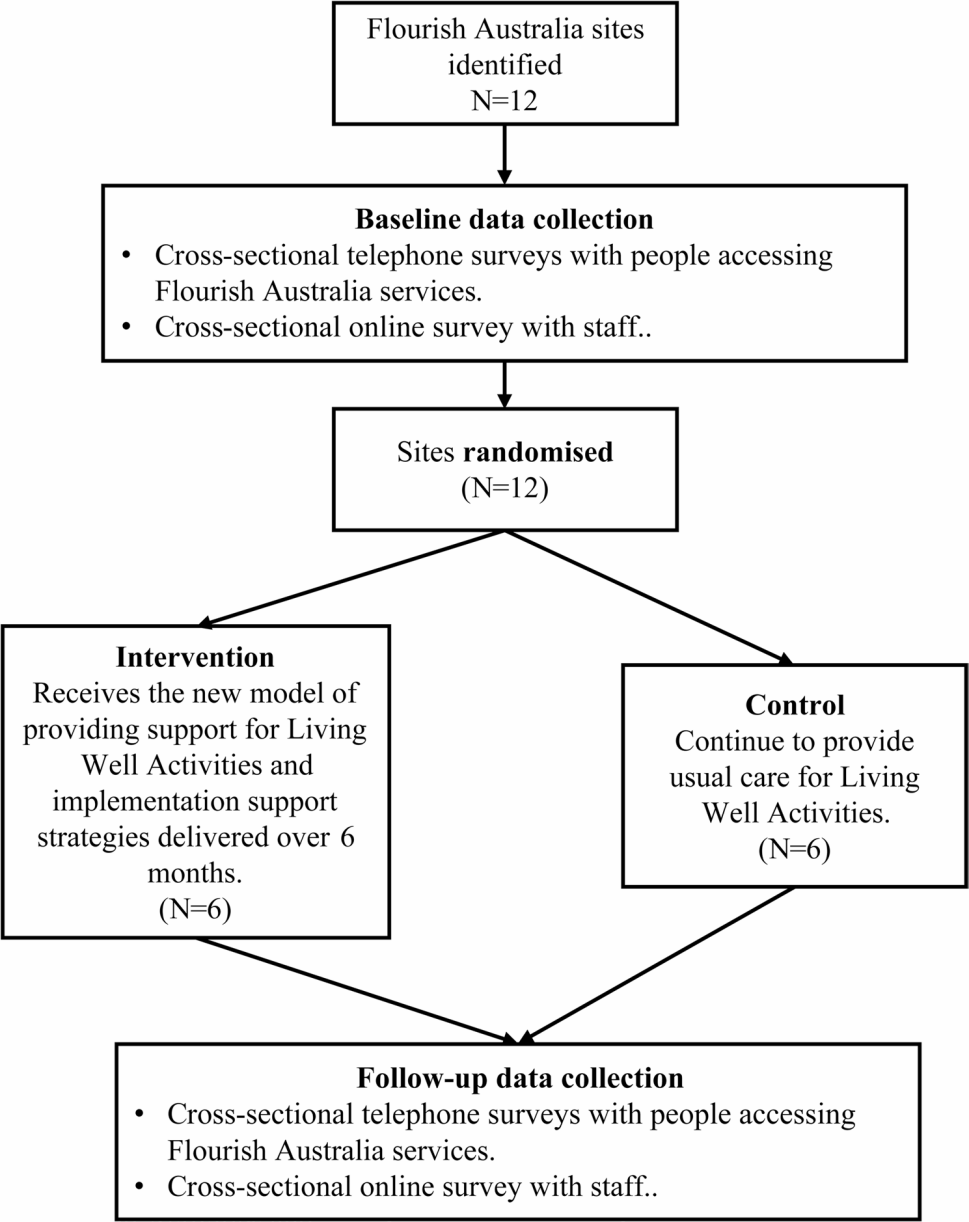
Study design

The study design is depicted in Fig. 1. Supplementary File 1 contains the SPIRIT schedule. After baseline survey data collection from all sites, sites will be randomised (1:1) to either a usual care control condition or a six-month practice change intervention. The intervention will comprise (1) a new model of providing support for Living Well Activities (i.e. preventive care), and (2) implementation strategies to support staff in delivering this care. The intervention will be implemented at the site level, independent of individual participation in data collection. Primary and secondary outcomes regarding receipt of preventive care will be measured through two rounds of independent cross-sectional telephone surveys with people accessing Flourish Australia services (baseline and post-intervention follow-up). Additional secondary outcomes regarding staff provision of care and perspectives of care for Living Well Activities, will be measured through two rounds of cross-sectional online surveys (baseline and post-intervention follow-up).

The study has been approved by the University of Newcastle Research Ethics Committee (Ref: H-2023-0321) and was prospectively registered with the Australian and New Zealand Clinical Trials Registry (ACTRN12625000322437).

#### Participant eligibility

##### Study sites

Twelve discrete sites will be recruited as clusters. Sites will be eligible if they have independent staff (i.e., no overlap of staff between sites) and offer support services lasting at least 6 weeks (to allow sufficient time for people accessing services to receive support for Living Well Activities). Sites currently involved in other major research initiatives will be excluded to avoid contamination. Approval to conduct the trial at each site will be obtained through the CMO’s regional managers.

##### People accessing supports

People currently receiving services at the participating CMO sites will be eligible to participate in the study survey if they meet inclusion criteria: (i) aged 18 years or older (ii) able to speak and read English (iii) sufficient mental and physical capacity to provide informed consent and complete the survey​. These criteria ensure that individuals can meaningfully participate in survey interviews and that their participation does not interfere with their care needs.

##### Staff

Support workers (frontline staff) from the CMO study sites will be recruited to participate in staff surveys. Inclusion criteria include: (i) age 18 years or older (ii) provide direct support to people receiving services at participating CMO sites.

### Procedures

#### Recruitment of people accessing supports

CMO administrative staff will facilitate participant recruitment to surveys by identifying those people accessing supports who meet the inclusion criteria and subsequently invite them to complete a ‘consent to contact’ form. *Invitation process*: Eligible people will be contacted via telephone, or email or physically approached by CMO staff, to provide them with brief plain-language information about the study purpose and what participation involves, as well as the detailed information statement. People accessing supports will be informed that participation is voluntary and independent of the routine support received from the service (i.e., opting out of the research will not affect any support they receive)​. *Consent to contact*: Interested people will be asked to complete a form (either online or printed) to share their contact details so they may be contacted by the research team. *Contact by the research team*: A member of the research team will then telephone each potential participant to confirm eligibility and invite them to participate in the study​. During this call, the researcher will review the study information again and obtain the participant’s informed consent verbally before proceeding with the baseline survey​. This three-step recruitment process will ensure that consent is well-informed and documented.

#### Recruitment of CMO staff

Approval will be obtained from the Flourish Australia managers at each site. Eligible staff will be contacted via email including an invitation to participate in the study survey, copy of the participant information statement, and an anonymous survey link by CMO administrative staff. Consent is implied by staff submitting a survey response.

### Randomisation and blinding

Twelve sites will be randomly allocated (cluster randomised) in a 1:1 ratio to either the intervention group or the control group (usual care) following completion of baseline surveys. An independent statistician not involved with participant recruitment will perform the randomisation using a computer-generated sequence, ensuring allocation concealment until the point of assignment. Due to the nature of the practice-change intervention, blinding is not possible for those delivering or receiving the intervention: CMO staff, managers, researchers coordinating the trial, and consumer participants in follow-up surveys will all be aware of whether their site is in the intervention or control condition​. The research team member conducting the primary outcome analyses will not be blinded to group assignment during the analysis phase​. To minimize bias, follow-up outcome data with people accessing Flourish Australia support will be collected by the research team members who will be blinded to the group assignment.

### Control

Services allocated to the control condition will follow usual practices in providing support for Living Well Activities. Staff will continue to use existing tools available within Flourish Australia’s Back on Track Health (BOTH) program to guide delivery of support for physical health (which may include Living Well Activities). These include the PhysiCards (a resource that can be used to identify health topics a person would like support for, including smoking, nutrition, alcohol and exercise), the Preventative Health Check-In (to support people to review their health and decide what services they would like to connect with), and a Camberwell Assessment of Need (CANSAS) inspired conversation tool (to reflect on life needs, including physical health, my food and diet, and alcohol) [36]. These tools are offered to consumers by support workers every six months. Goals and identified support needs may be recorded within each person’s Individual Recovery Plan (IRP). Our previous research with Flourish Australia indicates provision of preventive care is part of existing practice but is not routinely provided for all Living Well Activities [13].

### Intervention

A six-month practice change intervention will be delivered within the sites allocated to the intervention condition to increase provision of support for the Living Well Activities, including facilitating connections to support and resources. The intervention will be comprised of two components: [1] a new co-developed model of care ‘The Live Well and Flourish Framework’, and [2] implementation strategies to support delivery of this model of care. The intervention will be delivered at a site level to all staff and people supported, independent of participation in data collection surveys. The intervention will be aligned with the existing BOTH program and promote usage of currently available tools (as described above).

#### Co-development process

A project steering group was established at project commencement, with representation from the research team and Flourish Australia management to guide the intervention development and implementation. A series of three workshops were held to co-develop the model of care and strategies; attended by people Flourish Australia support, Flourish Australia staff and the research team. The process and outcomes of these workshops will be reported separately.

#### Model of care: ‘Live well and flourish framework’

**Fig. 2:**
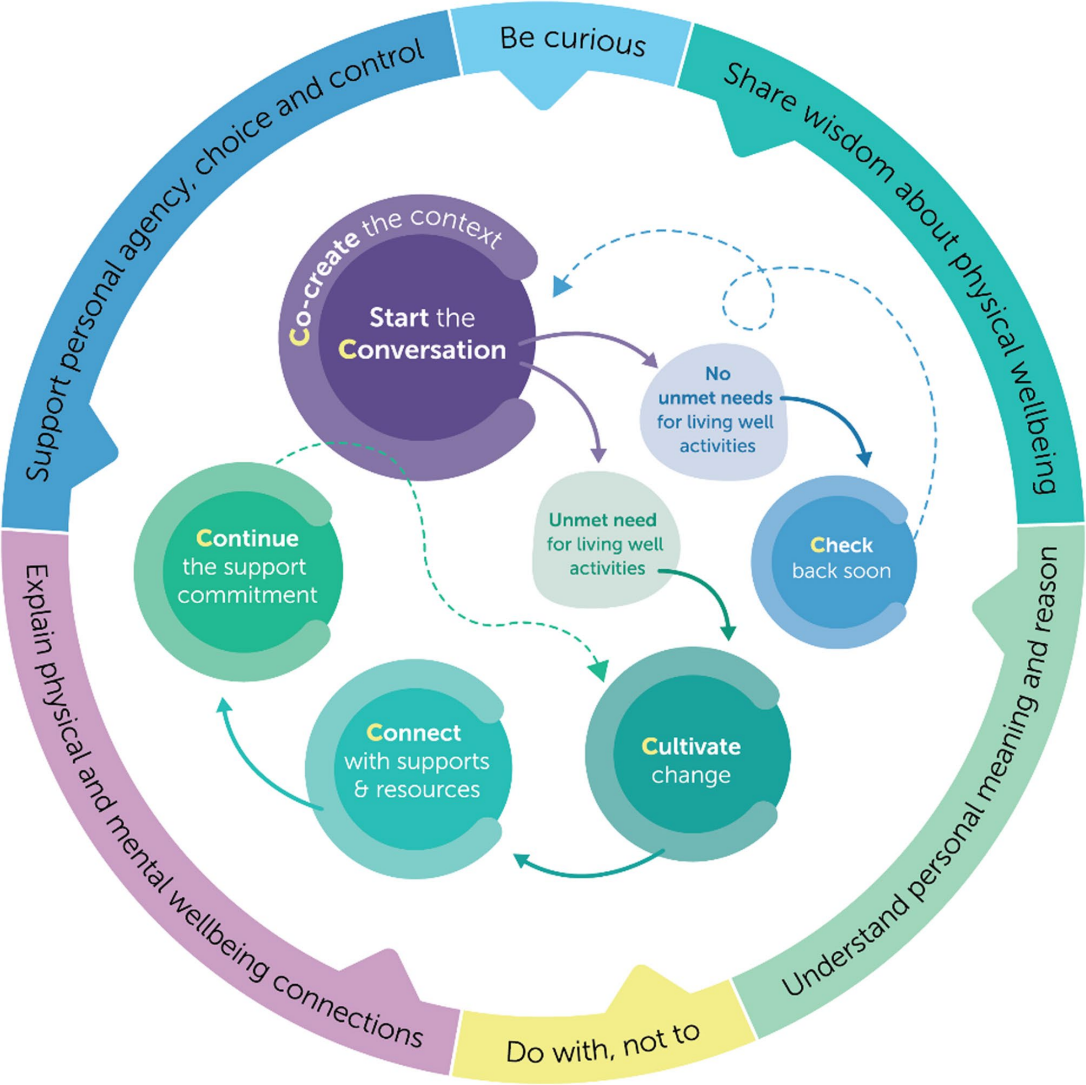
The Live Well & Flourish Framework

All staff providing direct support at the intervention sites will offer support for Living Well Activities, guided by the Live Well and Flourish Framework and supported by structured implementation strategies. The Framework consists of five ‘moments’ and six values that guide support, shown in Fig. 2 (see Supplementary Table 1 for definitions of the values). The five moments are:

1. *Co-create the context*: The staff member and the person supported agree they would like to discuss Living Well Activities and organise a time and place to do so.
2. *Start the conversation*: to understand what the person’s Living Well Activities look like now and if they have any unmet needs around these activities that they would like support for. Unmet needs are identified and recorded using the CANSAS tool. If the person does not have any unmet needs they would like support for, the staff member will check-in again in 6 months.
3. *Cultivate change*: The staff member and person supported work together to explore reasons for changing Living Well Activities, build motivation, and identify what changes the person would like to make. Existing tools may also be used (i.e., the PhysiCards and the Preventative Health–Check-in) and goals will be recorded in the IRPs.
4. *Connect with supports and resources*: The staff member and person supported decide together what support would be helpful for Living Well Activities. This may include accessing local services, providers or groups (e.g., health care provider, walking group, free population-level telephone coaching services including Quitline and Get Healthy Service,); resources (e.g., online information, recipe book); and/or support offered within Flourish Australia (e.g., cooking group, grocery support). The staff member will offer to facilitate access by considering what help or assistance the person may need and then actioning the connection e.g., helping the person to make an appointment, providing a website address with information about a support, or providing transport assistance. Connections made and support required will be recorded within IRPs.
5. *Continue the support commitment*: The staff member and person supported check-back in at agreed times to discuss what the person has been working on, any new actions the person might be taking or want to take, the supports they seek (if any), and celebrate achievements together.

#### Implementation strategies

Implementation strategies will be delivered at the intervention sites over six months to support staff with implementing the new Framework. The co-development process ensured the identified strategies addressed key barriers to care, are feasible and aligned with Cochrane review evidence [37–40].

1. *Leadership support and mandating change*: managers from the implementation sites will be invited to join a project steering group to guide and review implementation during the intervention period. Additional members will also be invited or consulted, including people accessing Flourish Australia services, staff representatives and project champions. Flourish Australia leaders, including the CEO, will reinforce and communicate support for the project and the importance of providing support in line with the Live Well and Flourish Framework e.g., via communication in regular staff meetings. At the commencement of the intervention, a project launch will be held by service leaders.
2. *Identify and prepare champions*: at each site, a staff member will be identified by Flourish Australia managers as the site champion. Their role will be to provide mentorship/support to staff, promote project activities, and collate and provide feedback to the project team. Champions will identify the opportunities to provide support and mentorship and seek feedback from both staff and people supported; with options developed as needed for each site (e.g., embedded in existing staff meetings, email communication, group reflective sessions).
3. *Staff training via educational outreach visits and online modules*: multi-mode (online and face-to-face) training will be provided. Face-to-face training in healthy conversation skills (4 h) will be offered to all support staff, and will be facilitated by either an experienced trainer or a Flourish Australia staff member trained via train-the-trainer sessions [28]. Five online modules of approximately 10–20 min each will be embedded in the organisation’s learning management system, with completion requested for all support staff. Each module focuses on one component of the Framework, detailing why it is important, what it includes, and how to implement it. Online modules will include evidence-based components e.g., scenarios, role plays and interactive elements [38].
4. *Educational materials for staff*: a Guide for staff on how to implement the Framework will be disseminated via hard-copy and existing electronic communication channels e.g., Microsoft Teams. The Guide will provide information about what the Framework is, how to provide support, and where this should be recorded in the electronic record system.
5. *Resources to guide support and involve people Flourish Australia supports*: Resources will include a conversation starter card tool; a pack of cards with prompts and topics to initiate conversations about Living Well Activities e.g., “what types of physical activities do you find fun?” (‘start the conversation’). Cards listing facts and myths about Living Well Activities will also be used to promote further conversation and build knowledge about how physical and mental wellbeing are connected e.g., “true or false: physical activity can be just as effective as medication in treating depression and anxiety” (‘cultivate change’). A Connections directory will be made available online which lists options for resources and supports, to help people in accessing services, with help from support workers (‘connect with supports and resources’). This directory may be used together during the support, or for the person supported to consider in their own time. Resources will be disseminated to staff via hard copies and existing electronic communication methods (e.g., Microsoft Teams) to share and use with the people they support. Posters and information in offices and centres will also help to prompt conversations about Living Well Activities, for both staff and people supported.
6. *Monitoring and ongoing feedback*: feedback will be collected from both staff and people supported on project progress via site champions (modes tailored to each site and supported by the champions e.g., meetings, a sealed box to collect anonymous feedback, and email communication). Data from electronic records will be used to monitor changes in completion rates of routine outcome measures and tools (e.g. inclusion of Living Well Activities in the IRP). Feedback will be summarised and reported on a regular basis to staff and people supported e.g., through monthly steering group meetings, in regular staff meetings (via champions), and project communication (e.g., emails, posters, and newsletters).

### Data collection methods

Data will be collected from people accessing Flourish Australia services and CMO staff at baseline and at follow-up after the intervention period (6 months following the intervention commencement) using repeated cross-sectional sampling. *People accessing services* will complete surveys administered via Computer-Assisted Telephone Interviews (CATI, programmed in REDCap), conducted by trained interviewers from the research team​. Using a CATI script, interviewers will ask participants a structured questionnaire about the support for Living Well Activities they have received, their current Living Well Activities, and perceptions of and satisfaction with support. Telephone interviews allow inclusion of participants who may have literacy or technology access barriers, and this method has been successfully used in similar trials to assess client-reported care receipt [29, 41, 42]​. *Staff* will be invited to complete a self-administered online survey (programmed in REDCap). At each data collection point, an email invitation (from the CMO management) will be sent to all eligible staff at the site with an anonymous survey link and study information statement​. Staff can complete the survey confidentially on their own device, providing information on the support they provide for Living Well Activities. Reminder emails will be sent to improve staff response rates.

### Measures

All survey instruments (for both people accessing supports and CMO staff) were developed specifically for this study. The content of the surveys was informed by prior studies on preventive care in mental health settings [16, 43], and co-developed with stakeholders including CMO managers, staff, people accessing Flourish Australia services and Flourish Australia Community Advisory Council members. This iterative development process involved reviewing the wording and relevance of each question to ensure they were understandable and appropriate for the CMO setting.

#### Primary outcome

The primary outcome for the trial will be the proportion of people accessing Flourish Australia services who report being offered (from the CMO) support to access services or resources for at least one Living Well Activity. Participants will be provided with a definition of the Living Well Activities and asked if *“Anytime within the last six months*,* has your support worker made any suggestions to visit or access services or suggested resources for any living well activities?*” with interviewers recording which Living Well Activity this was for, or “none” as appropriate.

#### Secondary outcomes

Secondary outcomes will be people accessing supports’ receipt and experience of support for Living Well Activities (five outcomes) as well as staff provision of and self-efficacy to provide this support (five outcomes); with questions informed by the Live Well and Flourish Framework.

##### People accessing Flourish Australia services receipt of support for living well activities

At each data collection time point (baseline and follow-up), participants will be asked to report whether they: (i) had a conversation with a support worker that led to identifying at least one Living Well Activity need; (ii) received support to develop plans for at least one Living Well Activity; (iii) made progress about actions/plans for at least one ‘living well activity’ need; and (iv) used at least one support or resource offered for a ‘living well activity’ (e.g. information, links to support groups, telephone support services such as the Get healthy Information and Coaching service, or specialist). Participants will also be asked (v) how satisfied they were with support received for Living Well Activities (rated on a three-point Likert scale: satisfied to dissatisfied).

##### Staff provision of support for living well activities

At both baseline and follow-up, staff will be asked to report the proportion of people they support (0% - not at all; 1–49% - less than half; 50–79% - more than half; 80–100% - most) for whom they have: (i) had conversations with (ii) developed plans or actions (iii) suggested resources/services to connect with (iv) checked back in with about their progress and continued to support needs after an initial conversation; for at least one Living Well Activity. Staff will also be asked to (v) rate their confidence to provide support for at least one Living Well Activity (10-point scale from 1 being ‘not at all confident’ through to 10 being ‘extremely confident’).

#### Current living well activities

Participants will self-report their engagement in each Living Well Activity. *Smoking and Vaping*: Using two items, participants will be asked about current tobacco smoking (including roll your own or cigars) and e-cigarette use (*daily*, *less than daily*, *no)*. *Nutrition (Fruit and Vegetable Intake)*: Daily consumption of fruits and vegetables will be measured by two items asking the number of servings of fruit and the number of servings of vegetables consumed on an average day. *Alcohol Use*: One item will report participants’ frequency of alcohol consumption in the past month *(Never*,* I don’t drink*,* none in the last month*,* once a month*,* 2–4 times a month/once a week*,* 2 to 3 times a week*,* 4 or more times a week)* [44]. *Physical Activity*: A single item will report the number of days in the last week they engaged in at least 30 min of moderate-intensity physical activity [45].

#### Sociodemographic characteristics

*People accessing supports*’ will self-report their age, gender identity, nationality, mental health condition, duration of engagement with the CMO, frequency of accessing support and usual location of support received. *CMO Staff* will self-report their age, gender, cultural identity, highest level of education attained, professional qualifications/background, duration of employment at the CMO, current employment status, current role or position title, number of individuals they typically support in an average week, and the approximate hours of support sessions they conduct per week.

### Process measures

The delivery and uptake of the implementation strategies will be recorded by the research team and champions in project logs, for example, steering group meetings held and attendance, staff completion of training, provision of feedback summaries, provision of project resources, and completion of relevant Flourish Australia tools (preventive health check-in, CANSAS, and IRP). In the follow-up surveys, the uptake of the strategies by staff will be measured e.g., use of the project resources. Staff will also report perceived acceptability, appropriateness and feasibility of the intervention and people supported will report acceptability of the model of care [46]. The project team will record any adaptations made to the intervention and contextual factors in the project logs. Qualitative interviews and/or focus groups with staff and people accessing Flourish Australia supports will be conducted at the intervention to collect additional process measures.

### Sample size

During each round of data collection (baseline and follow-up), approximately 370 people supported by Flourish Australia will be recruited to take part in the survey (185 intervention group, 185 control group). Assuming a baseline prevalence of the primary outcome of 50%, and an intra-class correlation of 0.05, this sample will provide an 80% power to detect an absolute 22.5% difference between the groups, with a type 1 error rate of 5%. Approximately 100 staff will be recruited in each round of data collection.

### Statistical analysis

Analyses will be conducted on an intention-to-treat basis. Characteristics of sites and people accessing supports will be summarised for each group using means and standard deviations (or medians and interquartile ranges) for continuous measures, and counts and percentages for categorical measures. Mixed effects logistic regression models will assess changes over time in the intervention compared to control group for primary and secondary outcomes. Models will include a fixed effect for group, random effect for cluster, and a fixed effect for individual to account for any repeated measures on people accessing support over time. Confounding variables such as age, gender and length of time accessing the CMO will also be adjusted for as fixed effects in the model. The threshold for statistical significance will be alpha = 0.05. Process and other measures will be summarised descriptively.

### Economic evaluation

The economic evaluation will comprise of (i) a costing analysis, (ii) a cost-consequence analysis using a scorecard approach to present costs in conjunction with the outcomes from the trial, and (iii) cost-effectiveness analysis, where the primary outcome will be incremental cost per percentage point increase in participants reporting receipt of support via an offer to facilitate access to resources Sensitivity analyses will be conducted to assess the impact of plausible variations in key input parameters, such as labour costs, informed by project logs.

Resource use and costs associated with the development and implementation of the model of care and related strategies will be prospectively identified, measured, and valued from a health service delivery perspective, in accordance with the Consolidated Health Economic Evaluation Reporting Standards (CHEERS) 2022 [47]. Data on resource use will be collected by the research team during the intervention period using structured project logs and qualitative interviews/focus groups. Cost categories will include: (1) implementation costs (e.g., materials, labour, and other direct expenses); (2) service delivery costs (e.g., staff time allocated to preventive care activities); and (3) opportunity costs borne by providers and service users. All costs will be reported in local currency and, where applicable, adjusted for inflation using appropriate indices. Costs related solely to research activities, such as data collection, will be excluded from the analysis.

## Discussion

People living with a mental health condition experience a greater burden of chronic disease morbidity and mortality worldwide, contributing to a reduced life expectancy [1]. Current recommendations suggest that mental health services routinely and systematically offer support for health risk factors which contribute to this inequity [5, 7]. An important and growing part of the mental health sector are CMO’s and previous research indicates that although they may be providing some support for health risk factors, there is scope for improvement [12, 13].

There is a need for interventions that assist CMO workers to provide routine and comprehensive care [10, 12, 13]. These interventions should be aligned with existing CMO models of care, which may be achieved through co-development with staff and people accessing services. This protocol describes a cluster-randomised controlled trial aiming to test the effectiveness of a practice change intervention to increase the provision of support for ‘Living Well Activities’ by support workers in a large CMO. This will include the delivery of a model of care, the ‘Live Well and Flourish Framework’, supported by evidence-based implementation strategies. Both the model of care and implementation strategies have been co-developed to ensure appropriateness, acceptability and feasibility within the mental health CMO setting. This protocol and the subsequent trial it describes will strengthen the research regarding effective implementation of interventions assisting CMO workers to support health behaviour change and promote physical and mental wellbeing. Outcomes of the trial will help inform future initiatives for such support in the mental health CMO sector, providing evidence of the cost-effectiveness of this support. These outcomes will inform both future research as well as the direction of practical policy and funding strategies.

## Supplementary Information

Supplementary Material 1.

## Data Availability

No datasets were generated or analysed during the current study.

## Abbreviations

CMO: Community Managed Organisation
NSW: New South Wales
AAR: Ask, advise, refer
5As: Assess, advise, agree, assist, arrange
NDIS: National Disability Insurance Scheme
SPIRIT: Standard Protocol Items: Recommendations for Interventional Trial
BOTH: Flourish Australia’s Back on Track Health Program
CANSAS: Camberwell Assessment of Need
IRP: Individual Recovery Plan
CATI: Computer-Assisted Telephone Interviews
CHEERS: Consolidated Health Economic Evaluation Reporting Standards
